# Associations between Adherence to the Mediterranean Diet and Lifestyle Assessed with the MEDLIFE Index among the Working Population

**DOI:** 10.3390/ijerph15102126

**Published:** 2018-09-27

**Authors:** Sandra Pavičić Žeželj, Gordana Kenđel Jovanović, Nataša Dragaš Zubalj, Vladimir Mićović, Željko Sesar

**Affiliations:** 1 Health Ecology Department, University of Rijeka, Faculty of Medicine, Braće Branchetta 20, Rijeka 51000, Croatia; ravnatelj@zzjzpgz.hr; 2 Department of Health Ecology, Teaching Institute of Public Health of Primorsko-goranska County, Krešimirova 52a, Rijeka 51000, Croatia; gordana.kendel-jovanovic@zzjzpgz.hr (G.K.J.); natasa.dragas-zubalj@zzjzpgz.hr (N.D.Z.); 3 Faculty of Maritime Studies, University of Rijeka, Studentska ulica 2, Rijeka 51000, Croatia; sesar.ordinacija@gmail.com

**Keywords:** cardiovascular diseases, lifestyle, Mediterranean diet, MEDLIFE index, workers

## Abstract

The adherence to the Mediterranean diet is beneficial for cardiovascular diseases prevention. The study aim is to use Mediterranean lifestyle (MEDLIFE) questionnaire for estimation of Mediterranean lifestyle habits among the working population and to establish MEDLIFE score correlation with the risk factors for cardiovascular diseases. In the study has participated 366 workers from Croatia, which fulfilled MEDLIFE and validated food frequency questionnaire (FFQ) questionnaire. The multivariate logistic regression was performed to evaluate the association between MEDLIFE index, workers’ obesity and cardiovascular diseases risk. The lowest adherence to Mediterranean lifestyle was associated to younger, low education, body fat above acceptable ranges and unfavorable lipid profile. Significant association to Mediterranean lifestyle was more among women (*p* = 0.002), middle aged (*p* = 0.02), highly physically active (*p* = 0.009) and those who play collective sports >2 h/w (*p* = 0.001), having body fat within acceptable range (*p* = 0.003), total cholesterol less (*p* = 0.03) and high-density lipoproteins (HDL-C) (*p* = 0.04) more than recommended. Inverse significant association was for high educational level (*p* = 0.02). The Mediterranean lifestyle adherence is associated to lower risk factors for cardiovascular diseases among studied working population. MEDLIFE index revealed that physical activity and conviviality are better ingrained among younger population but not the Mediterranean diet.

## 1. Introduction

Public health intervention programs aimed for workers’ wellness have been successfully conducted at workplace, since it represents a good setting due to population’s large sample which can be repeatedly surveyed [1]. Before the workplace public health intervention program, the thorough and validated prior assessment of workers’ health and lifestyle habits should be conducted. According to the WHO, Croatia adulthood obesity prevalence forecasts (2010–2030) predict that in 2020, 35% of men and 42% of women will be obese which represent significant increase from the current 20.1% and 20.6%, respectively [2]. Today, there is a great focus on the benefits of the traditional Mediterranean diet (MD) because of its many proven health welfare [3,4], for successful workplace health intervention results for cardiovascular diseases (CVD) incidence and development of prevention [5]. MD showed to be long adhered to and more appealing than other dietary choices [6]. Other lifestyle behavior’s like physical activity and some psychosocial determinants, particularly job stress, unhealthy or insufficient resting time, depression or anxiety [7] influence the workers’ illness. Croatia is one of seven countries that have nominated Mediterranean diet for inscription on the Representative List of the Intangible Cultural Heritage of Humanity at UNESCO (No. 00884) in 2013, so it is necessary to evaluate the MD existence and to monitor MD pattern changes in Croatia [8]. Besides that, it is valuable to evaluate Mediterranean lifestyle, since it has significant influence on health [9] and it represents a significant part and heritage of Croatian coastal region, even though it has not been evaluated yet. Recently, Spanish investigators designed, developed and validated a questionnaire that can determine individual’s adherence to the Mediterranean lifestyle by the diet and physical activity patterns assessment called MEDLIFE [10], which also includes social interactions typical for Mediterranean lifestyle, such as sociability, siesta, cultural and gastronomic factors. MEDLIFE questionnaire could successfully estimate all lifestyle behaviors and could be used in epidemiological and clinical studies as a valid instrument to measure Mediterranean lifestyle adherence [10]. No previous studies, besides Spain study of MEDLIFE authors [10], have used MEDLIFE index in assessing Mediterranean diet and lifestyle. Croatia has been classified as a country with high risk for CVD [11], and CVD mortality rates are almost twice as high as the EU average, which points to the possible shortcomings in health care delivery and public health interventions [12].

The aim of this study is to use MEDLIFE questionnaire for estimation of Mediterranean lifestyle habits of workers of Primorsko-goranska County in Croatia. The goal is to establish the correlation of the MEDLIFE score with the risk factors for CVD among the working population.

## 2. Materials and Methods

### 2.1. Study Participants

The study population consisted of 366 workers, out of 547 employed at oil and gas company, with sedentary type of occupations. They approved to participate after the presentation of the public health intervention program for improving lifestyle habits by diet and physical activity, organized by the Teaching Institute of Public Health of Primorsko-goranska County. The study was previously approved by the Teaching Institute of Public Health of Primorsko-goranska County Ethical committee. Workers filled out the whole questionnaire, done health examination and entered in the study after having filled out the written informed consent.

### 2.2. Participants’ Anthropometric and Cardiovascular Disease Variables

The health examination included body anthropometric and composition measurements and blood sample for lipid profile assessment. Workers’ body weight and composition (fat mass, lean mass and bone mass) was measured by using the electronic scale (Tanita BC-544^®^, Tokyo, Japan). Height was measured by the mechanical stadiometer platform (Seca^®^ 274, Hamburg, Germany). Body mass index (BMI) was calculated as the body weight in kilograms divided by the square of height in meters. Workers’ waist circumferences (WC) measurements to the nearest 0.1 cm were measured with non-elastic tape. Blood samples were collected in the morning by experienced nurse after at least 12 h fasting and analyzed within one day after sampling. Lipid profile included levels of triglycerides (TG), total cholesterol, cholesterol linked to high-density lipoproteins (HDL-C) and it was analyzed using spectrophotometric methods. The fraction of cholesterol linked to low-density lipoproteins (LDL-C) was calculated using the Friedewald equation [13]. Workers were classified according to recommended classification of BMI, WC and lipid profile regarding European Guidelines on cardiovascular disease prevention in clinical practice [14] into within and above recommendations. Also, workers were classified by lower and upper end of acceptable body fat, sex and age range for adults older than 18 years [15] into within and above acceptable range.

### 2.3. The Questionnaire

The study questionnaire was divided in two parts and designed according to MEDLIFE questionnaire. The first part contained sociodemographic questions about age, sex, education level based on the finished school, physical activity, leisure lifestyle and particular dietary habits. Physical activity habits were estimated using International Physical Activity Questionnaire (IPAQ) [16]. Leisure lifestyle habits were estimated from questions on time spent sitting (h/d), screen watching (TV, video, computer) (h/d), sleeping (h/d), napping (siesta) (h/d) and socializing with friends (h/d), according to weekdays and weekends. In addition, participants were asked about dietary supplements and medications consumption, about particular dietary habits like nibbling between meals, adding salt and sugar to meals and beverages and their preferences regarding whole grain products.

### 2.4. Dietary Assessment

Dietary intakes and habits were assessed in the second part of the questionnaire, i.e., validated FFQ for Mediterranean diet-based populations [17], which were adapted and used for the diet evaluation of Croatian students [18] and women population [19]. By the registered nutritionist assistance, the participants have noted their usual consumption frequency of 97 offered foods over the preceding year, considering seasonal variations, in the offered choices from the options ‘never’ to ‘more than three times a day’. For each food offered, the serving sizes were specified as small, medium and large which was accompanied by photos.

### 2.5. Mediterranean Lifestyle (MEDLIFE) Index

The principles of the Mediterranean Diet Pyramid: a lifestyle for today, that proposed by the Spanish Mediterranean Diet Foundation, was the basis for the MEDLIFE index design [20]. A total of twenty-eight items of MEDLIFE index, divided in three blocks, interpreted the MD pyramid and scored each item with 0 or 1 [10]. First MEDLIFE block evaluates fifteen items on food consumption frequency in the offered servings, while the second block evaluates seven items on Mediterranean dietary habits. The third block evaluates six items on physical activity, rest, social habits and conviviality. Overall MEDLIFE index scored from 0 which signified as worst, to 28 designated as best. According to provided MEDLIFE index scores of the participants, they were divided into working groups for lifestyle, diet and physical activity improvements based on education intervention and counselling by the specialists of the Teaching Institute of Public Health of Primorsko-goranska County.

### 2.6. Statistical Analysis

Differences in continuous variables between MEDLIFE index subgroups among men and women separately were evaluated with the Student’s *t*-test and ANOVA analyses for normally distributed variables, while for the non-parametric variables were used the Mann-Whitney test and Kruskal-Wallis test. Frequencies are presented as absolute numbers and percentages, while the continuous variables are presented as means and standard deviations. To assess the association between individual MEDLIFE component score to total MEDLIFE index the chi-square test was used for nominal variables. Each participants’ MEDLIFE components was tested for association to their overall MEDLIFE index with Spearman rank correlation. Based on the obtained adherence score for characteristics, nutrition and lifestyle of each participant, they were divided into quartiles. The multivariate logistic regression, after adjustment for age, sex, smoking and physical activity, was performed to evaluate the association between workers’ characteristics with adherence to MD and lifestyle considered as highest quartile of MEDLIFE score, meaning high adherence to Mediterranean lifestyle. All statistical tests were made for 2-tailed hypotheses and *p* < 0.05 was regarded as statistically significant. All statistical analyses were conducted using software system Statistica, version 13, (TIBCO Software Inc., Palo Alto, CA, USA, 2017).

## 3. Results

In study participated almost equally men (48.4%) and women (51.6%), where women had significantly slightly better adherence score than men (14.5 and 13.1 respectively; *p* = 0.002) (Table 1).

Significantly better adherence score was noticed among middle age participants (*p* = 0.04), while younger participants had the worst MEDLIFE score. There was a significantly better score among highly physically active participants (*p* = 0.03), those with normal weight (*p* = 0.001), with body fat within acceptable range (*p* = 0.02) and among those with recommended HDL-C level (*p* = 0.02). As there were significantly more participants that were highly educated, they were expected to have better MEDLIFE score because of possible better nutrition knowledge, but the results showed that medium educational level group had better score than others, but with no significance. There was no significant difference in MEDLIFE score by smoking habits and recommendation classification of WC, blood cholesterol, LDL-C and TG. Almost three times more participants had unfavourable total and LDL-C (*p* = 0.01), and there were significantly more of participants with favourable HDL-C (*p* = 0.04). Those with adverse lipid profile had lower MEDLIFE score but not significantly different to those with favourable lipid profile. Considering that in our study participated 366 workers with average MEDLIFE score 13.8, the MEDLIFE score classification was into quartile groups as follows: Q1 (8–11), Q2 (12–13), Q3 (14–15) and Q4 (16–19). When it comes to MEDLIFE, 16.4% of participants (Q1) had low adherence, 63.1% had medium adherence (Q2 and Q3) and 20.5% higher adherence (Q4). 

Table 2 shows proportion of participants when scored 1 point for each of 28 MEDLIFE component, also each MEDLIFE component of the participants mean intakes after age-, sex- and energy adjustment. They are presented as servings per week or per day, and as hours per day, week or weekend concerning rest, social habits and conviviality, across quartile distribution. High percentage of participants (96.7%) scored 1 point for potato consumption, olive oil (92.6%), cereals (91.0%), herbs (88.5%), fish/seafood (69.7%), white meat (64.8%) and for water or tea (64.8%) consumption. About half of participants scored 1 point for processed meat (50.0%), eggs and legumes (54.1%), and fruits consumption (51.2%). About third of participants scored 1 point for vegetables (33.6%), low-fat dairy products (27.9%), nuts (24.6%) and snacks (22.2%). Participants scored worst for sweets consumption (9.8%) and red meat consumption (14.8%), while 17.2% of participants scored for one or two glasses of wine per day. Significantly more restricted salt (48.4%) than sugar (16.4%), and 84.4% of them reported to limit their snacking between meals. Only 19.7% reported whole grains preference. Regarding the resting hours, social habits and conviviality, all participants reported to sleep 6 to 8 h per day, 61.5% socialized more than 2 h per week with friends, while 23.8% of them took a siesta during weekends. Third of participants (27.0%) were physically active more than 2.5 h/week or 30 min/day and 30.3% were involved in collective sports (jogging, soccer, basketball etc.). Significant difference between participants’ quartiles was for processed meat (*p* < 0.000), snacks (*p* < 0.000), nuts (*p* < 0.000), vegetables (*p* < 0.000) and salting meal limitation (*p* < 0.000). Spearman correlation coefficients between MEDLIFE components and overall MEDLIFE index (Table 2), done for content validity among study participants, has revealed medium correlation for vegetables (0.66), limiting salt (0.53), nuts (0.52), and weak correlation for processed meat (0.50), snacks (0.48), fish/seafood (0.42), legumes (0.39), and fruits (0.35).

We found significant association to Mediterranean lifestyle as high adherence (highest quartile Q4) among women (OR = 1.3, 95% CI = 1.1–1.5), among highly physically active (OR = 1.3, 95% CI = 1.1–1.7) and those playing collective sports more than 2 h per week (OR = 1.3, 95% CI = 1.1–1.6) (Table 3).

Significant association to high adherence was noted among middle aged workers compared to other age groups (OR = 1.3, 95% CI = 1.1–1.7), for those having body fat within acceptable range (OR = 1.3, 95% CI = 1.1–1.6), having total cholesterol lower (OR = 1.2, 95% CI = 1.0–1.4) and HDL-C (OR = 1.9, 95% CI = 1.0–3.6) higher than recommended. Those with normal weight had 37% more odds to had MD and Mediterranean lifestyle compared to those being obese (OR = 1.4, 95% CI = 0.8–2.3). Inverse significant association was found among those with high educational level (OR = 0.6, 95% CI = 0.4–0.9). Those who never smoked and former smokers had 15% more odds to adhere to Mediterranean lifestyle (OR = 1.2, 95% CI = 0.9–1.4; OR = 1.2, 95% CI = 1.0–1.40, respectively). To verify relation of MD to MEDLIFE index, the participants’ diet has been correlated and evaluated with a previously validated and used Mediterranean Diet Score index (MDS) [21] to MEDLIFE index. Out of total 9 point of MDS index score, our participants scored on average 5.0 (SD 1.7), meaning that they had diet that moderately adhered to MD. The MDS index significantly correlated to MEDLIFE index (ρ = 0.70; *p* < 0.0001) and showed strong connection to MD habits (Table 3).

## 4. Discussion

To our knowledge, this is the first Croatian study on Mediterranean lifestyle that, beside MD habits, evaluates habits of physical activity, adequate rest in form of siesta and social engagements such as socializing with friends or family. This study revealed that two thirds of our participants socialized with friends more than 2 h per week, third were physically active and siesta was practiced by one quarter of participants. MEDLIFE index showed that siesta was not significant part of Mediterranean way of life of our participants but conviviality or playing collective sports was significant. Our participants were slightly more physically active and more socialized, they had more adequate rest, but two times less in the form of siesta and two times more spent time screen watching than the Spanish workers’ that evaluated with MEDLIFE index [10]. Highly physically active participants adhered significantly better to the Mediterranean lifestyle, especially those who played collective sports more than 2 h per week. Those findings could be explained by the younger age of our participants, since the average age of highly active participants was 30.3 years, while average age of participants with medium and low physical activity habits was 38.3 years. It is assumed that physically active lifestyle is adopted as a healthy way of life, because of physical activity health messages to which the younger generations are more exposed to, through their education and electronic media usage. Younger participants could have more free leisure time compared to older work associates, and more time to take care of themselves, and they chose to spend it by being physically active. In adverse, younger participants had more unhealthy dietary habits which weakly adhered to MD. It is assumed that younger participants disregard diet quality, cooking and MD heritage which represents potential unhealthy dietary habits with possible effects on health at older age, and it is necessary for public health nutrition actions to focus more on younger populations. Recent study on adherence to MD among 2768 inhabitants of southern Croatia [22] also revealed diminishing adherence to MD among younger generations, and authors also emphasized the public health priority in order to improve dietary habits with emphasis on MD due to its importance of cultural heritage.

Most of our participants had dietary habits that moderately adhered to MD, but there were almost twice more participants with dietary habits close to MD than those not adhering to MD. Italians also showed that their younger inhabitants of Southern Italy progressively abound traditional MD habits [23], which they explained with economic costs of healthier foods included in MD, hypothesizing that noticed food pattern shift is due to higher availability of lower nutritional quality foods accompanied with healthy foods higher prices. Croatian food prices are a bit lower than European means, lower than Italian and very similar to Spain and Portugal prices [24]. The previously mentioned Italian study [23] also revealed that MD was directly associated with education, more among those with medium education level, which is like our results. South West England workplace nutrition intervention with MD [25] also found MD better connection with higher education. The authors, based on their findings, suggested that future interventions should promote more legumes, fish and olive oil and to decrease intake of red meat and poultry which could be used in our settings, due to similar food groups’ consumptions results of our participants. Our workers consumed insufficiently legumes, fruits and vegetables, with high prevalence of sweets, snacks and red meat consumption, which is more similar to western-like dietary pattern. It results that the plant-based promotion is a profound outset for future Croatian public health nutrition promotion. The food group consumption of the participants had similarities to recent Spain study [26], where the researchers highlighted lower adherence to MD noticed in different parts of Spain, which is in line with the high prevalence of hypertension representing increased CVD risk. Croatia has been classified as a high-risk CVD risk country [11], and by evaluating the participants’ lipid profile we could better arrange their later nutrition and lifestyle education in order to prevent CVD development. Cardiovascular diseases are the main cause of premature death among working European population and WHO European Region is setting a target to reduce CVD premature deaths by 1.5% annually until 2020 [27], so with public health intervention programs that use MEDLIFE index could be useful for CVD prevention. MEDLIFE index showed that MD and lifestyle is significantly connected to acceptable range fat content which could be explained with participants’ high physical activity. Our participants mostly tend to have unfavorable lipid profile, higher total cholesterol level, but significantly high proportion of participants had good HDL-C level which could be due to participants’ younger age. Therefore, it is necessary to monitor youngsters’ health and lifestyle to prevent later possible CVD events, along with promotion of Mediterranean diet and lifestyle. It is worthy to mention that high proportion of participants had recommended daily olive oil and weekly fish intake and those MD foods are recognized in preventing CVD risks [3]. The study done on 1284 hospitalized patients due to acute or chronic ischemic heart disease in hospitals across Croatia showed that patients with chronic heart disease had more MD habits, concluding that MD is associated with reduced risk of developing cardiovascular disease [28]. Those participants with unfavorable lipid profile had lower adherence to MD and lifestyle, which expose them more to CVD risk, and our results are similar to the results of lipid profile of Spanish workers [29]. Those workers with western-like diet characteristics had lover HDL-C levels while those with diet close to MD had higher HDL-C levels, which is also similar to our findings. In fact, the participants with favorable HDL-C level had significantly 93% better odds to adhere to Mediterranean lifestyle.

Our study had some limitations that include relatively small number of participants, possible recall bias, and a lack of other possible confounders such as marital status, number of family members and income values that could affect dietary and lifestyle habits as well as the adherence to Mediterranean diet and lifestyle. 

The strengths of our study findings rely on similarities to other studies among working population on adherence to MD and lipid profile, showing that adults from northern Mediterranean part of Croatia have similar Mediterranean lifestyle habits to those from other parts of Mediterranean [10,22,23,26]. It is revealed that Mediterranean diet and lifestyle is more present among women, middle aged and those with medium education level. Croatian dietary study among women [19] showed that by aging women tend to improve their diet quality. Therefore, as women have substantial influence on family dietary habits, it would be significant for public health dietary messages to address more to middle aged women. Physical activity and non-smoking as healthy way of life were also better connected to Mediterranean lifestyle, likewise participants’ better health condition. Although there were confirmed previous similar findings that younger population diminish Mediterranean dietary pattern, MEDLIFE index revealed that Mediterranean lifestyle like physical activity and conviviality is better ingrained among younger participants, which presents MEDLIFE index a good tool for revealing significant health confounders. It is shown that MEDLFE index is simple to use, valid and significant for revealing the Mediterranean lifestyle adherence, also in revealing the MD and lifestyle association to CVD biomarkers, and for lifestyle assessment in public health intervention programs.

## 5. Conclusions

It can be concluded that Mediterranean lifestyle adherence is associated to lower risk factors for CVD among studied participants. The study results have revealed that future public health messages should be focused more on younger generations promoting more Mediterranean diet and lifestyle, using nutrition education by utilizing more electronic media. Croatian study on students’ nutrition knowledge [18] showed significant influence of better nutrition knowledge on better diet quality, so it is important to provide better nutrition education to younger generations. In addition, by promoting Mediterranean lifestyle, not only MD, public health programs should encourage employees to promote and implement Mediterranean lifestyle among their employees and directly participate in CVD prevention. The workplace is proven to be a good setting to implement healthy lifestyle through nutrition and lifestyle education, so by using MEDLIFE index as prior assessment tool it is possible to arrange better and target intervention program among working population.

## Figures and Tables

**Table 1:** Mediterranean Lifestyle adherence assessed by MEDLIFE of 366 participants across their characteristics.

Characteristics	*n* (%)	Total Mean (SD)	Medlife Mean (SD)	*p*
Sex				
Men	177 (48.4)		13.1 (2.8)	0.002 *
Women	189 (51.6)		14.5 (2.5)	
Age (years)		37.2 (8.6)		
Age groups <30 years	93 (25.4)	26.8 (1.4)	13.0 (2.3)	0.04 *
30–39 years	141 (38.5)	35.1 (2.5)	14.0 (2.6)	
40–49 years	87 (23.8)	44.0 (2.7)	14.5 (2.2)	
≥50 years	45 (12.3)	52.3 (1.9)	13.7 (2.3)	
Education level				
Low	21 (5.7)		11.7 (2.6)	0.22
Medium	39 (10.7)		14.5 (2.9)	
High	306 (83.6)		13.9 (2.3)	
Smoking habits				
Never	183 (50.0)		14.1 (2.2)	0.10
Former	75 (20.5)		14.2 (2.5)	
Current	108 (29.5)		13.2 (2.7)	
Physical activity (MET-h/w)		42.5 (10.0)		
Low	105 (28.7)	36.8 (11.8)	13.1 (2.5)	0.03 *
Medium	183 (50.0)	43.4 (6.6)	13.7 (2.0)	
High	78 (21.3)	48.2 (10.3)	15.1 (2.9)	
BMI (kg/m^2^)		24.7 (3.0)		
Normal weight	165 (45.1)	22.0 (1.9)	14.8 (2.6)	0.001 *
Overweight	189 (51.6)	26.6 (1.3)	13.0 (2.1)	
Obese	12 (3.3)	31.3 (0.2)	13.3 (0.8)	
WC (cm)		84.0 (9.2)		
<94 men; ≥80 women	264 (72.1)	81.0 (7.4)	14.0 (2.4)	0.44
≥94 men; ≥80 women	102 (27.9)	91.7 (9.1)	13.3 (2.5)	
Body fat (%)		24.9 (6.7)		
within acceptable range	90 (24.6)	18.2 (3.7)	15.0 (3.0)	0.02 *
>acceptable range	276 (75.4)	27.0 (6.1)	13.4 (2.1)	
Cholesterol (mmol/L)		5.6 (1.0)		
<5.0 mmol/L	117 (31.9)	4.5 (0.3)	14.6 (3.0)	0.19
≥5.0 mmol/L	249 (68.1)	6.1 (0.7)	13.5 (2.1)	
HDL-C (mmol/L)		1.5 (0.3)		
≥1.0 mmol/L men; ≥1.2 mmol/L women	357 (97.5)	1.6 (0.3)	13.9 (2.4)	0.02 *
<1.0 mmol/L men; <1.2 mmol/L women	18 (2.5)	0.9 (0.2)	11.0 (0.9)	
LDL-C (mmol/L)		3.4 (0.9)		
<3.0 mmol/L	138 (37.7)	2.5 (0.3)	14.3 (2.9)	0.33
≥3.0 mmol/L	228 (62.3)	4.0 (0.6)	13.5 (2.1)	
TG (mmol/L)		1.4 (0.7)		
<1.7 mmol/L	270 (73.8)	1.0 (0.3)	14.0 (2.3)	0.34
≥1.7 mmol/L	96 (26.2)	2.4 (0.5)	13.4 (2.8)	

* *p* < 0.05. MET, metabolic equivalent of task; BMI, body mass index; WC, waist circumference; HDL-C, cholesterol linked to high-density lipoproteins; LDL-C, cholesterol linked to low-density lipoproteins; TG, triglycerides.

**Table 2:** Age-, sex- and energy-adjusted Mediterranean Lifestyle index components mean intakes and correlations according to quartile distribution among 366 participants.

MEDLIFE Components	*n* (%)	Q1 (*n* =48)		Q2 (*n* = 138)		Q3 (*n* = 93)		Q4 (*n* = 87)		*p*	Spearman Rank ρ
		Mean	95% CI	Mean	95% CI	Mean	95% CI	Mean	95% CI		
Servings/week											
Sweets	36 (9.8)	5.0	3.1, 6.0	5.0	4.1, 5.9	4.8	3.6, 6.0	4.0	2.1, 7.5	0.25	−0.16
Red meat	54 (14.8)	3.7	1.7, 5.1	2.9	2.6, 3.2	2.8	2.5, 3.1	2.9	1.5, 4.8	0.01	−0.21
Processed meat	183 (50.0)	2.5	0.3, 4.2	1.4	1.1, 1.7	1.1	0.7, 1.4	0.6	0.0, 2.2	<0.000 *	−0.45
Eggs	198 (54.1)	0.8	0.0, 3.6	0.6	0.4, 0.7	0.6	0.4, 0.7	0.9	0.1, 2.1	0.09	0.13
Legumes	198 (54.1)	0.6	0.0, 1.4	0.7	0.4, 0.9	1.0	0.7, 1.2	1.8	0.4, 3.5	<0.000 *	0.39
White meat	237 (64.8)	0.9	0.0, 2.9	1.0	0.9, 1.2	1.2	0.9, 1.5	1.1	0.8, 2.9	0.84	−0.03
Fish/seafood	255 (69.7)	1.5	0.8, 2.8	1.9	1.5, 2.3	2.9	2.3, 3.5	3.7	0.4, 10.8	0.000 *	0.42
Potatoes	354 (96.7)	1.2	0.0, 4.1	1.1	0.9, 1.2	0.9	0.7, 1.0	1.2	0.1, 4.2	0.96	−0.17
Snacks	105 (22.2)	0.0	0.0, 0.0	0.2	0.0, 0.4	0.2	0.0, 0.4	1.1	0.0, 2.5	<0.000 *	0.48
Tea	237 (64.8)	6.3	5.6, 8.8	6.9	6.0, 7.4	7.8	7.1, 8.5	6.5	5.6, 8.8	0.03	0.03
Servings/day											
Dairy products	102 (27.9)	0.9	0.1, 2.0	0.9	0.8, 1.1	0.8	0.6, 0.9	0.7	0.1, 1.6	0.45	−0.13
Nuts	90 (24.6)	0.2	0.0, 1.2	0.3	0.2, 0.5	0.7	0.4, 0.1	1.3	0.1, 2.7	<0.000 *	0.52
Herbs	324 (88.5)	1.1	0.2, 1.9	0.7	0.5, 0.8	1.5	1.2, 1.8	1.2	0.1, 1.6	0.21	0.17
Fruit	243 (51.2)	0.6	0.1, 1.6	1.8	1.6, 2.1	2.6	2.1, 3.1	4.0	2.3, 4.1	0.05	0.35
Vegetables	123 (33.6)	1.2	0.4, 2.8	0.5	0.4, 0.6	0.7	0.5, 0.8	4.1	2.4, 6.1	<0.000*	0.66
Olive oil	339 (92.6)	0.6	0.1, 0.9	5.9	5.2, 6.6	5.9	5.1, 6.7	0.4	0.2, 1.7	0.52	0.09
Cereals	333 (91.0)	7.8	2.5, 11.2	0.7	0.5, 0.8	1.5	1.2, 1.8	5.4	1.5, 7.3	0.09	−0.20
Wine	63 (17.2)	0.3	0.0, 0.7	0.3	0.2, 0.4	0.3	0.1, 0.4	0.4	0.0, 1.5	0.35	0.09
Whole grains preference **	72 (19.7)	0.0	0.0, 0.0	23.9	11.1, 36.7	23.3	7.3, 39.4	20.7	5.0, 36.4	0.10	0.08
Limit salt **	177 (48.4)	6.3	7.1, 19.6	37.0	22.5, 51.5	56.7	37.9, 75.5	79.3	63.6, 95.0	<0.000 *	0.53
Limit nibbling **	309 (84.4)	81.3	59.8, 92.7	87.0	76.8, 97.1	80.0	64.8, 95.2	86.2	72.7, 99.6	0.91	0.01
Limit sugar **	60 (16.4)	0.0	0.0, 0.0	15.2	4.4, 26.0	10.0	1.4, 21.4	34.5	16.1, 52.9	0.005 *	0.18
Physical activity **	99 (27.0)	31.3	5.7, 56.7	19.6	7.7, 31.5	26.7	9.9, 43.5	38.0	19.2, 56.7	0.24	0.12
Siesta/Nap **	87 (23.8)	12.5	5.7, 30.7	23.9	11.1, 36.7	23.3	7.3, 39.4	31.0	13.1, 48.9	0.38	0.17
Hours of sleep/d	366 (100.0)	7.6	6.0, 8.0	7.3	7.1, 7.5	7.1	6.8, 7.4	6.9	6.0, 8.0	0.008 *	−0.23
Screen watching (h/d)	84 (22.9)	2.1	0.3, 4.0	1.9	1.5, 2.3	1.5	1.1, 1.9	1.9	0.8, 4.0	0.33	−0.05
Socialize (h/weekend)	225 (61.5)	1.3	0.0, 4.0	1.7	1.2, 2.1	1.9	1.3, 2.6	1.3	0.0, 4.0	0.29	0.02
Collective sports (h/week)	111 (30.3)	0.3	0.0, 4.0	0.8	0.4, 1.3	0.8	0.3, 1.2	1.3	0.0, 4.0	0.02 *	0.27

* *p* < 0.05. ** Categorical variables are presented as the percentage of people who reach 1 point and 95% confidence interval.

**Table 3:** Participants’ characteristics by MEDLIFE quartiles and multiple logistic regression analysis of variables associated with high adherence to MEDLIFE (Q1–Q3 vs. Q4).

Characteristics	MEDLIFE Score				*p*	High Adherence OR (95% CI)	*p*
	Q1	Q2	Q3	Q4			
Sex					0.23		
Men	30 (62.5)	75 (54.3)	39 (41.9)	33 (37.9)		1	
Women	18 (37.5)	63 (45.7)	54 (58.1)	54 (62.1)		1.3 (1.1, 1.5)	0.002 *
Age groups					0.02 *		
<30 y	24 (50.0)	45 (32.6)	9 (9.6)	15 (17.2)		1	
30–39 y	15 (31.2)	45 (32.6)	48 (51.6)	33 (37.9)		1.2 (1.0, 1.5)	0.09
40–49 y	0 (0.0)	39 (28.3)	18 (19.4)	30 (34.5)		1.3 (1.1, 1.7)	0.02 *
≥50 y	9 (18.8)	9 (6.5)	18 (19.4)	9 (10.4)		1.2 (0.9, 1.5)	0.31
Education level					0.25		
Low	6 (12.5)	12 (8.7)	3 (3.2)	0 (0.0)		1	
Medium	0 (0.0)	21 (15.2)	6 (6.5)	12 (13.8)		1.1 (0.9, 1.4)	0.36
High	42 (87.5)	105 (76.1)	84 (90.3)	75 (86.2)		0.6 (0.4, 0.9)	0.02 *
Smoking habits					0.15		
Current	24 (50.0)	48 (34.8)	18 (19.4)	18 (20.7)		1	
Former	12 (25.0)	18 (13.0)	18 (19.4)	24 (27.6)		1.2 (0.9, 1.4)	0.17
Never	12 (25.0)	72 (52.2)	57 (61.2)	45 (51.7)		1.2 (1.0, 1.4)	0.10
Physical activity					0.07		
Low	18 (37.5)	45 (32.6)	30 (32.2)	12 (13.8)		1	
Medium	18 (37.5)	81 (58.7)	42 (45.2)	42 (48.3)		1.1 (0.9, 1.4)	0.16
High	12 (25.0)	12 (8.7)	21 (22.6)	33 (37.9)		1.3 (1.1, 1.7)	0.009 *
Siesta/nap during weekend					0.49		
No	42 (87.5)	102 (73.9)	75 (80.6)	60 (69.0)		1	
Yes	6 (12.5)	36 (26.1)	18 (19.4)	27 (31.0)		1.1 (0.9, 1.3)	0.23
Socializing with friends					0.21		
<2 h/w	30 (62.5)	48 (34.8)	33 (35.5)	30 (34.5)		1	
≥2 h/w	18 (37.5)	90 (65.2)	60 (64.5)	57 (65.5)		1.1 (0.9–1.3)	0.21
Collective sports					0.000 *		
<2 h/w	45 (93.7)	102 (73.9)	63 (67.3)	45 (51.7)		1	
≥2 h/w	3 (6.3)	36 (26.1)	30 (32.2)	42 (48.3)		1.3 (1.1–1.6)	0.001 *
BMI Obese	0 (0.0)	9 (6.5)	3 (3.2)	0 (0.0)		1	
Overweight	33 (68.8)	75 (54.4)	48 (51.6)	33 (38.0)	0.25	0.9 (0.6–1.6)	0.82
Normal weight	15 (31.2)	54 (39.1)	42 (45.2)	54 (62.1)		1.4 (0.8–2.3)	0.24
WC					0.79		
≥94 cm men; ≥80 cm women	18 (37.5)	36 (26.1)	27 (29.0)	21 (24.1)		1	
<94 cm men; ≥ 80 cm women	30 (62.5)	102 (73.9)	66 (71.0)	66 (75.9)		1.1 (1.0–1.4)	0.15
Body fat (%)					0.000 *		
>acceptable range	27 (56.2)	126 (91.3)	75 (80.6)	45 (51.7)		1	
within acceptable range	21 (43.8)	12 (8.7)	18 (19.4)	42 (48.3)		1.3 (1.1–1.6)	0.003 *
Cholesterol					0.01 *		
≥5.0 mmol/L	27 (56.2)	102 (73.9)	75 (80.6)	45 (51.7)		1	
<5.0 mmol/L	21 (43.8)	36 (26.1)	18 (19.4)	42 (48.3)		1.2 (1.0–1.4)	0.03 *
HDL-C <1.0 mmol/L men <1.2 mmol/L women	6 (12.5)	3 (2.2)	0 (0.0)	0 (0.0)	0.04 *	1	
≥1.0 mmol/L men ≥1.2 mmol/L women	42 (87.5)	135 (97.8)	93 (100.0)	87 (100.0)		1.9 (1.0–3.6)	0.04 *
LDL-C ≥3.0 mmol/L	21 (43.8)	96 (69.6)	66 (71.0)	45 (51.7)	0.12	1	
<3.0 mmol/L	27 (56.2)	42 (30.4)	27 (29.0)	42 (48.3)		1.1 (1.0–1.3)	0.09
TG					0.55		
≥1.7 mmol/L	18 (37.5)	30 (21.7)	21 (22.6)	27 (31.0)		1	
<1.7 mmol/L	30 (62.5)	108 (78.3)	72 (77.4)	60 (69.0)		1.1 (0.9–1.3)	0.25

* *p* < 0.05. BMI, body mass index; WC, waist circumference; HDL, cholesterol linked to high-density lipoproteins; LDL, cholesterol linked to low-density lipoproteins; TG, triglycerides.
